# Femur-tibia angle and patella-tibia angle: new indicators for diagnosing anterior cruciate ligament tears in magnetic resonance imaging

**DOI:** 10.1186/s13102-022-00462-w

**Published:** 2022-04-13

**Authors:** Zeng Li, Mengyuan Li, Yan Du, Mo Zhang, Hai Jiang, Ruiying Zhang, Yuanchen Ma, Qiujian Zheng

**Affiliations:** 1 Department of Orthopedics, Guangdong Provincial People’s Hospital, Guangdong Academy of Medical Sciences, Guangzhou, 510080 China; 2 Clinical Research Unit, Obstetrics and Gynecology Hospital of Fudan University, Shanghai, 200011 China

**Keywords:** Anterior cruciate ligament, Femur-tibia angle, Patella-tibia angle, Magnetic resonance imaging, Ligament tears

## Abstract

**Background:**

Femur-tibia angle (FTA) and patella-tibia angle (PTA) are two MRI measurements that reflect the rotation of the knee joint. The purposes of this study were to assess whether FTA and PTA are associated with ACL tear and to explore their roles in ACL tear diagnosis.

**Methods:**

FTA, PTA, ACL angle and anterior tibial subluxation were compared between the two matched groups: ACL tear group and control group (each n = 20). Diagnostic performance was evaluated in a consecutive 120-patient cohort who underwent MR imaging of the knee and subsequently had arthroscopy. Different measurements were assessed by area under the curve (AUC) of receiver operating characteristic (ROC) curve.

**Results:**

FTA and PTA increased significantly in ACL tears group when compared to the control group (4.79 and 7.36 degrees, respectively, *p* < 0.05). In distinguishing complete ACL tear, ACL angle had the highest AUC of 0.906 while AUC of PTA and FTA were 0.849 and 0.809. The cutoff of FTA was 80 degrees with a sensitivity of 82% and specificity of 68%, while the cutoff of PTA was 91 degrees with a sensitivity of 82% and specificity of 74%. In distinguishing partial ACL tear, FTA and PTA had the highest AUCs of 0.847 and 0.813, respectively. The calculated cutoff of FTA was 84 degrees with a sensitivity of 90% and specificity of 81%, while the cutoff of PTA was 92 degrees with a sensitivity of 80% and specificity of 77%.

**Conclusion:**

FTA and PTA increased when ACL tears and they might be valuable in diagnosing ACL tears, especially in distinguishing partial ACL tear from intact ACL.

## Background

Anterior cruciate ligament (ACL) is the most commonly injured ligament of the knee joint. Magnetic resonance imaging (MRI) of the knee has been used as a diagnostic tool for over thirty years and widely used in clinical evaluation of ACL [1, 2]. It has been reported that the sensitivities of MRI in diagnosing complete ACL tears are 90–95% while the specificities are 95–100% [2]. Although the accuracy in diagnosing complete ACL tears by MRI is relative high, partial ACL tears are poorly diagnosed. In clinical practice, partial ACL tears are often indistinguishable from intact ACL and the accuracy of MRI diagnosis was only 25% to 53%, which may cause misdiagnosis and affect treatment decision-making [3, 4].

**Fig. 1:**
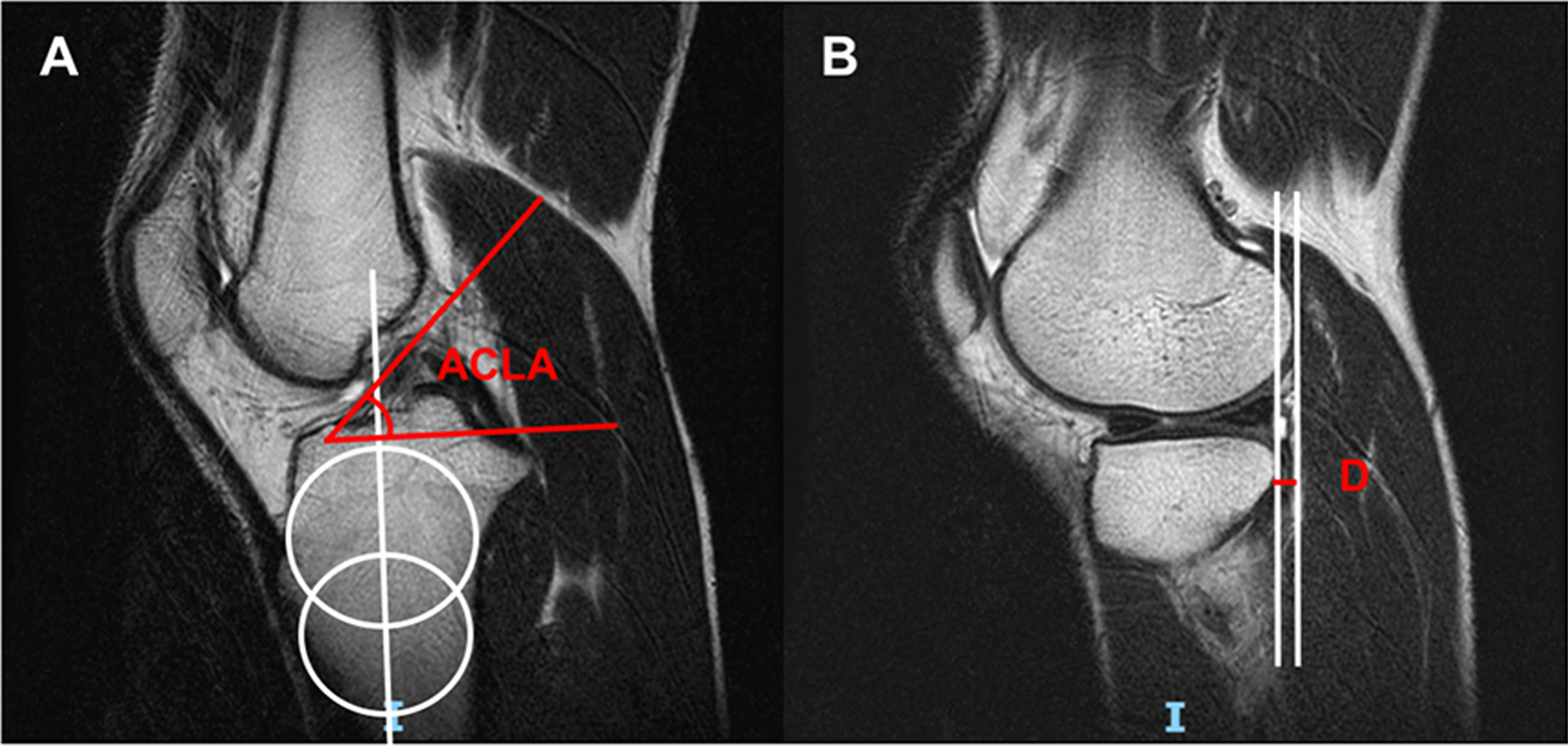
The measurements of ACL angle and anterior tibial subluxation. **A** ACL angle; **B** anterior tibial subluxation. (ACLA: ACL angle; D: distance of anterior tibial subluxation)

ACL extends from the medial aspect of the lateral femoral condyle and inserts at the anterior intercondylar region of the proximal tibia, which can be seen in the oblique sagittal imaging of MRI [5]. The frequently used methods for identifying torn ACL include direct and indirect signs [6]. The primary direct sign is the continuity and signals of ACL. Normal ACL remains a well-defined band-like structure in MRI. When the ACL is torn, it will lose its continuity or become tortuous with abnormal signals, or even disappear in chronic tears [7]. One of the indirect signs is ACL angle which reflects the tension of ACL and decreases when the ACL is torn (Fig. 1A) [8]. Another important sign is anterior tibial subluxation which increases with the increased clinical laxity of the knee joint after ACL tears (Fig. 1B) [9]. However, the above mentioned signs seem not available for partial ACL tears. Subtle increased signal intensity in ACL is often the only sign in partial ACL tears, which is indistinguishable from intact ACL. The ACL angle or anterior tibial subluxation can also be normal, which lead to the low accuracy of diagnosis of partial ACL tears [10]. Therefore, novel MRI indicators are needed to improve the accuracy of diagnosis on partial ACL tears to help preoperative treatment decision-making.

When the ACL is torn, it will not only cause obvious anterior tibial subluxation, but also result in significant internal rotation of the tibia [11]. In physical examination, pivot shift test has been commonly used to assess the rotatory laxity of knee joint caused by ACL tear [12]. Quantitative pivot shift test has also been validated in clinical trials [13]. In the present study, two quantitative parameters were introduced, femur-tibia angle (FTA) and patella-tibia angle (PTA), to measure the rotation of knee joint. The purposes of this study were to assess whether FTA and PTA are associated with ACL tear and to explore their roles in ACL tear diagnosis.

## Methods

This was a retrospective study which approved by the Research and Ethics Institutional Committee of Guangdong Provincial People's Hospital (KY-Q-2022-070-01) and conducted according to the Declaration of Helsinki. All methods were carried out in accordance with relevant guidelines and regulations. The need for informed consent was waived by Research and Ethics Institutional Committee because it was a retrospective analysis from the hospital database.

### Study design

The study retrospectively reviewed the cases of primary arthroscopic knee surgery from the Arthroscopy Database at Guangdong Provincial People's Hospital (GDPH). All the patients have taken MR imaging in GDPH before knee arthroscopy.

FTA, PTA and previously reported measurements (introduced in the MRI Measurements part) were compared between the two groups: ACL tear group and control group (each n = 20) [8, 9]. The patients were extracted from January 2018 to December 2019. Patients aged more than 18 years old with arthroscopically confirmed complete ACL tears or no ACL injury were included. Patients with patellofemoral instability, patella alta or baja, other ligament injury (collateral ligament or posterior cruciate ligament), osteoarthritis (Outerbridge grade > 2) were excluded [14, 15]. After extracted, the patients were matched in sex, age, body mass index (BMI), side and time to surgery.

Diagnostic performance of the measurements was evaluated in a 120-patient cohort who underwent MR imaging of the knee and subsequently had arthroscopy. The patients were included consecutively from January 2020 to December 2020 and no specific exclusion criteria were applied in this part.

### MRI measurements

All patients receiving imaging at our institution had undergone MRI at 1.5 T (Siemens Healthcare) with 3 imaging planes (axial, coronal, and sagittal). Slice thickness was 3 mm for each plane. Scans were performed in the supine position with relaxed quadriceps. All images were reviewed and analyzed using electronic radiology patient archiving and communication system (PACS) [16].

**Fig. 2:**
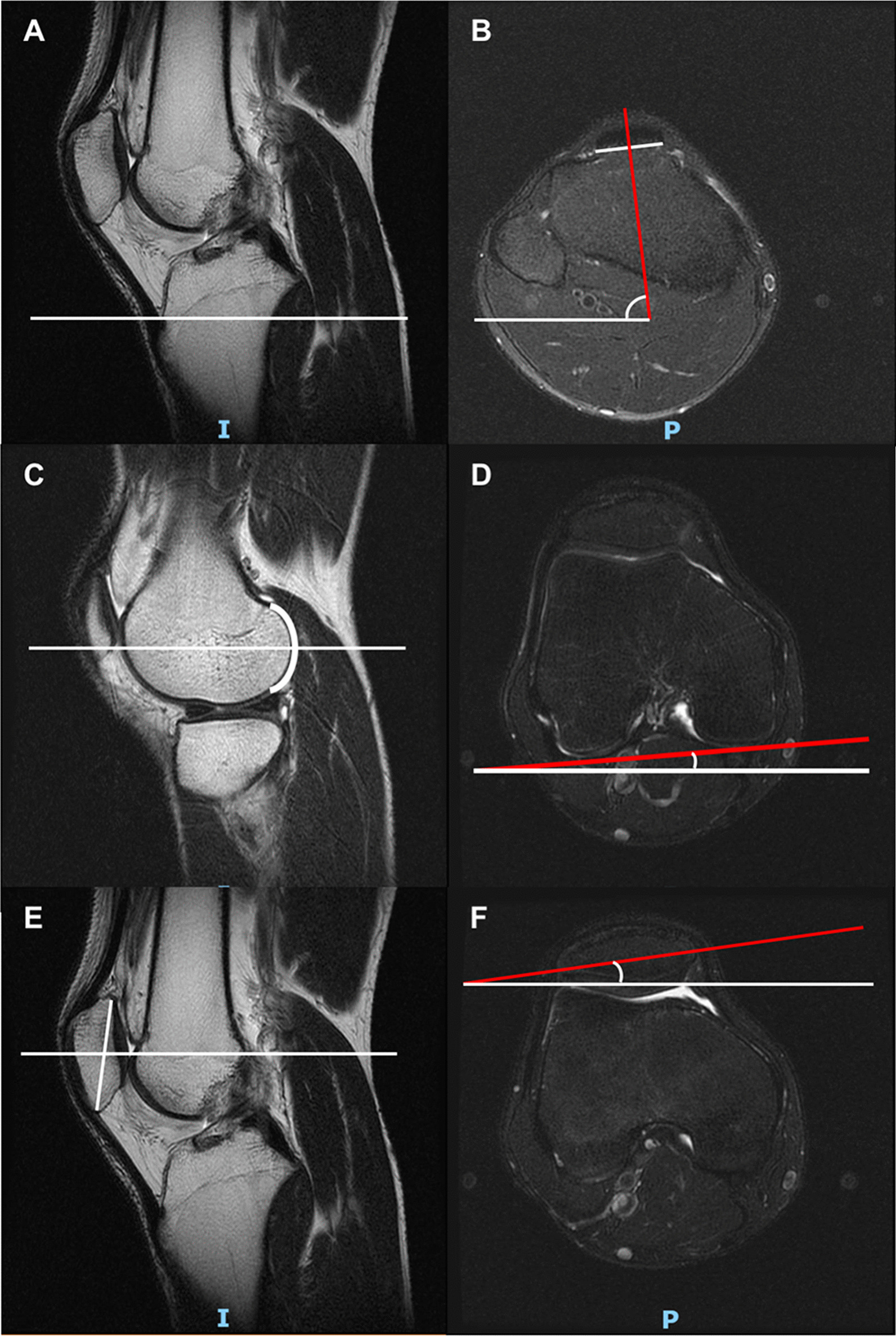
The measurement of tibial angle, femoral angle and patellar angle. **A**, **B**. the tibial angle; **C**, **D** the femoral angle; **E**, **F** the patellar angle. The angle is defined as positive when it directs laterally, and as negative when it directs medially

**Fig. 3:**
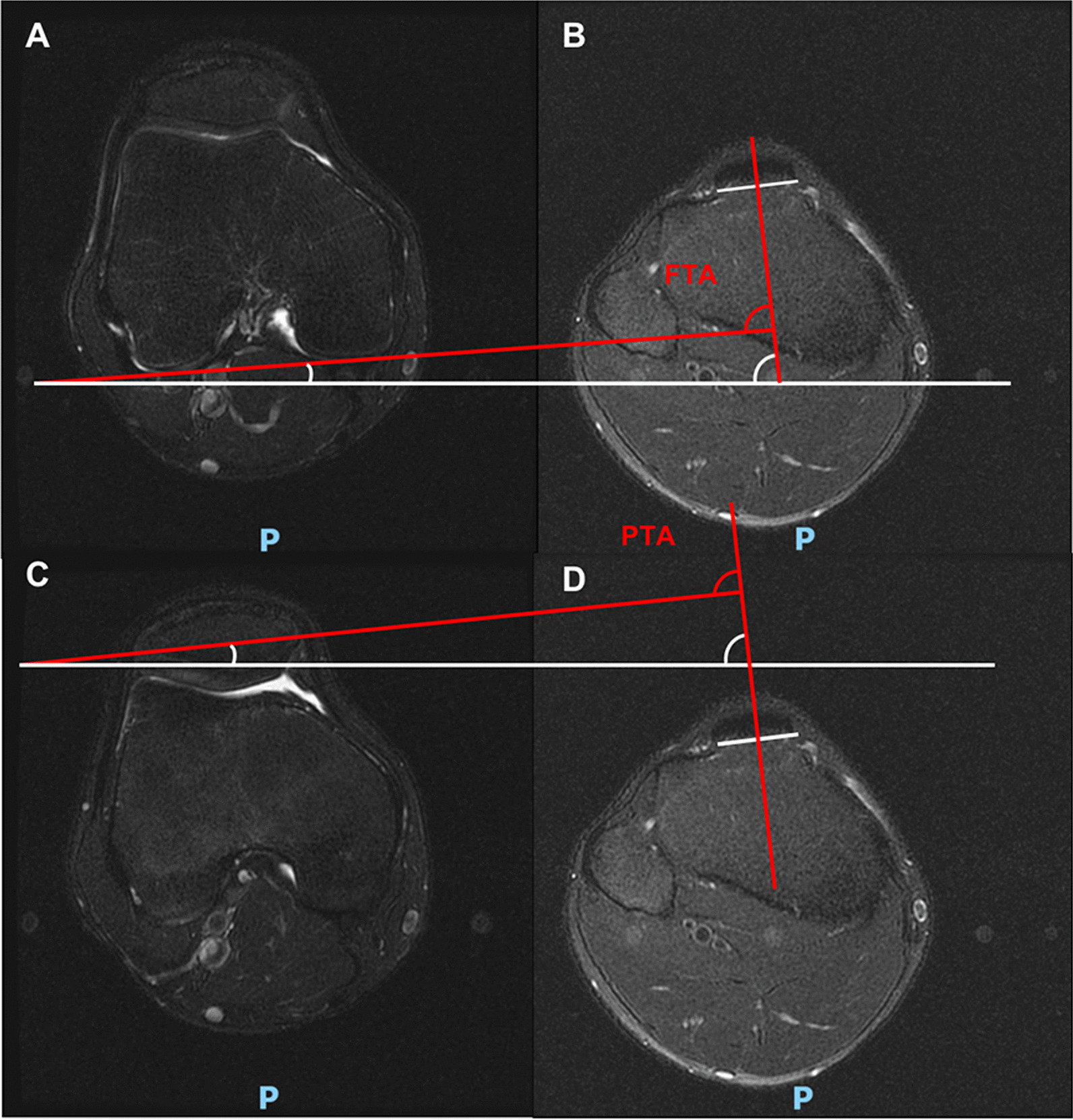
The measurement of FTA and PTA. **A**, **B** FTA is the tibial angle minus the femoral angle. **C**, **D** PTA is the tibial angle minus the patellar angle. (FTA: femur-tibia angle, PTA: patella-tibia angle)

To measure the rotation between tibia and femur or patella, the study developed two indicators: femur-tibia angle (FTA) and patella-tibia angle (PTA). FTA indicates the rotation between tibia and femur, while PTA presents the rotation between tibia and patella. FTA or PTA are calculated with the help of three angles: tibial angle, femoral angle and patellar angle. For tibial angle, the axial plane of patellar tendon insertion in tibia is defined at the level of midsagittal plane (Fig. 2A). The tibial angle is the intersection between the line perpendicular to patellar tendon insertion and the horizontal line (Fig. 2B). For femoral angle, the axial plane of femur is defined at the level of the most posterior aspects in midsagittal plane of lateral femoral condyle (Fig. 2C). The femoral angle is the intersection between the line of the posterior femoral condyles and the horizontal line (Fig. 2D). For patellar angle, the axial plane of patella was defined in middle of the patella at the level of midsagittal plane (Fig. 2E). The patellar angle is the intersection between the long axis line of patella and the horizontal line (Fig. 2F). The angles are defined as positive when directed laterally, and as negative when directed medially. Therefore, FTA is the tibial angle minus the femoral angle and PTA is the tibial angle minus the patellar angle (Fig. 3).

ACL angle and distance of anterior tibial subluxation (D) are used to assess the ACL function. Patella shift, lateral patellofemoral angle (LPFA) and patellar tilt are used to evaluate the patellar alignment change [17–20]. Descriptions of these measurements are given in Table 1. All measurements are defined as positive when it directed laterally, and as negative when it directed medially. The measurements were performed by two authors independently. The mean value was used if the differences between the angles were less than 5 degrees or the distances were less than 2 mm. The reliability of different observers was tested by concordance rates and interclass correlation value.

**Table 1:** Descriptions of measurements of ACL and patella

	Definition
ACL angle (°)	The angle between the tangential line of ACL and the line perpendicular to the long axis of the tibia in sagittal plane
D (mm)	The distance between the posterior aspects of lateral femoral condyle and tibial plateau in sagittal plane
Patella shift (mm)	The distance between the eminence of patella and the bottom of femoral trochlea at the level of line of posterior femoral condyles in coronal plane
LPFA (°)	The angle between the line of the femoral trochlea eminences and the tangential lines of the lateral facet of the patella in coronal plane
Patellar tilt (°)	The angle between the line of the posterior femoral condyles and long axis line of patella in coronal plane

ACL, anterior cruciate ligament; D, distance of anterior tibial subluxation; LPFA, lateral patellofemoral angle

### Statistics

Quantitative variables were presented as mean and standard deviation (SD). For normally distributed continuous variables, unpaired Student’s t-test was used to compare the differences between the two groups. For not normally distributed continuous variables, Mann–Whitney U-test was used to compare the differences between two groups. Categorical variables were presented as number and percentage, and were compared using Chi-square test or Fisher’s exact test when appropriate. Receiver operating characteristic (ROC) curve was used to determine the sensitivity and specificity of ACL, D, FTA and PTA. The area under the curve (AUC) and its 95% confidence interval (CI) were calculated. The AUC was tested by a two-sided binomial z test. The optimal cutoff value was determined at the maximal Youden index [21]. Interclass correlation (ICC) was tested between different observers. A p-value of less than 0.05 was considered as statistically significant. Data were analyzed using IBM SPSS version 23.0.

## Results

**Fig. 4:**
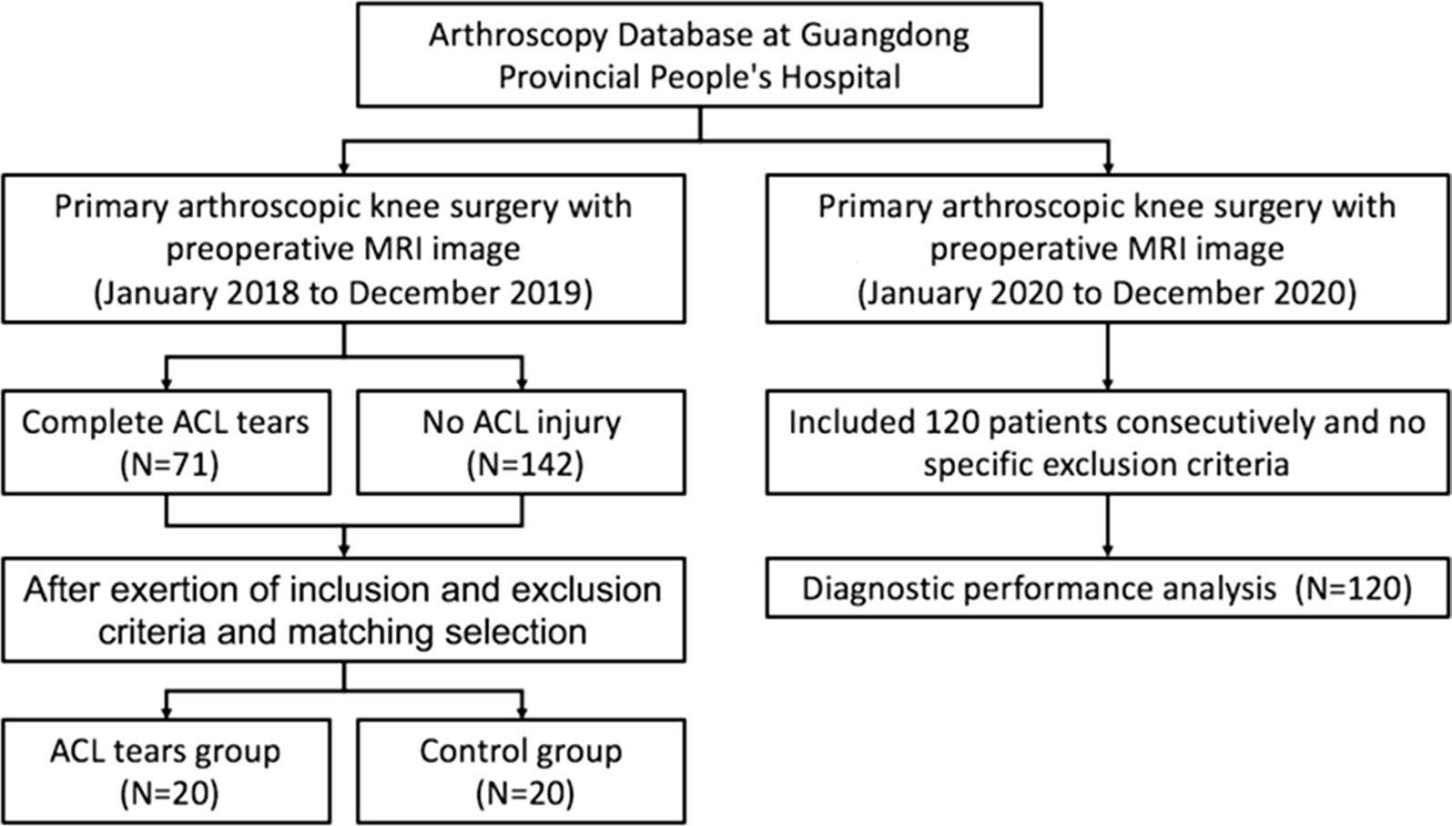
Flowchart of the study participants. (ACL: anterior cruciate ligament)

Forty patients were included for the matched comparisons (20 for ACL tears group and 20 for control group) (Fig. 4). Each group comprised 10 women and 10 men. After matching, no significant differences were observed between the two groups regarding age, BMI, injury side, and time from injury to surgery (*p* > 0.05) (Table 2).

**Table 2:** Demographic and medical conditions in ACL tear group and control group

	ACL tear group	Control group	*P*
*n*	20	20	–
Sex (male)	10	10	1.000
Age (year)	29.40 (9.16)	32.78 (8.42)	0.355
BMI (kg/m^2^)	22.49 (2.31)	23.44 (3.68)	0.430
Side (left)	10	10	1.000
Time (month)	7.67 (4.88)	6.88 (3.60)	0.883

ACL, anterior cruciate ligament; BMI, body mass index

In comparisons, there were significant differences in the measurements of ACL angle, D, FTA and PTA (*p* < 0.05) between the two groups. For ACL angle, the average angle was 49.55 degree in the control group, significantly higher than that of the ACL tears group (39.71 degree, *p* < 0.001). However, the ACL was not detected in 40% (8/20) cases of ACL tears group and in 5% (1/20) of the control group. D was also different between the two groups. The ACL tears group had a greater anterior tibial subluxation than the control group (*p* = 0.004). For the two measurements, FTA and PTA indicated the rotation of the knee. In ACL group, the FTA and PTA had 4.79 and 7.36 degrees more than that of the control group (*p* = 0.022 and < 0.001, respectively). Power analysis indicated that FTA and PTA had the power of 76.98% and 99.06%, respectively. No significant differences were found in the measurements regarding the alignment of patellofemoral joint, including patella shift, patellar tilt and LPFA between the two groups (*p* > 0.05 for all) (Table 3).

**Table 3:** Measurements in ACL tear group and control group

	ACL tear group	Control group	*P*
n	20	20	–
ACL angle (°)	39.71 (7.57)	49.55 (6.83)	< 0.001
D (mm)	4.90 (2.55)	2.94 (3.33)	0.004
Patella shift (mm)	0.79 (3.49)	0.71 (2.60)	0.937
LPFA (°)	10.3 (4.93)	11.1 (5.94)	0.534
Patellar tilt (°)	− 7.00 (4.72)	− 4.43 (5.58)	0.124
FTA (°)	85.70 (6.60)	80.91 (6.11)	0.022
PTA (°)	92.70 (5.32)	85.34 (6.30)	< 0.001

ACL, anterior cruciate ligament; D, distance of anterior tibial subluxation; LPFA, lateral patellofemoral angle; FTA, femur-tibia angle; PTA, patella-tibia angle

To further test the diagnostic performance of ACL angle, D, FTA and PTA, 120 consecutive patients aged more than 18 years old were included. There were 70 men and 50 women with an average age of 35.02 ± 8.34 years old. Fifty-five patients were diagnosed as complete ACL tear, while 11 as partial ACL tear and 54 as intact ACL. All these cases were diagnosed by arthroscopy.

**Fig. 5:**
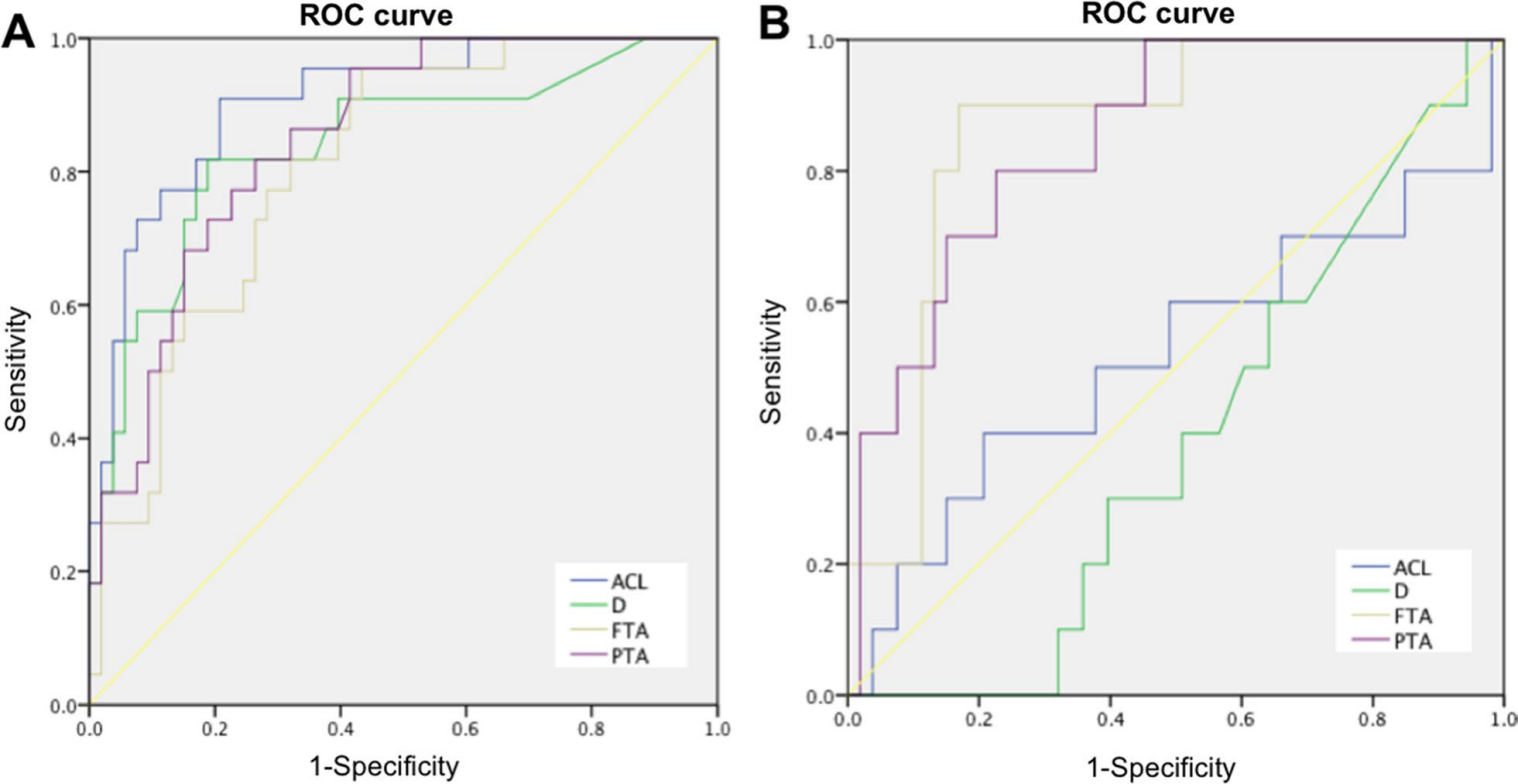
ROC curves in diagnosing test. **A** ROC curves in distinguishing complete ACL tear with intact ACL. **B** ROC curves in distinguishing partial ACL tear with intact ACL. (ACL angle: anterior cruciate ligament angle; D: distance of anterior tibial subluxation; FTA: femur-tibia angle; PTA: patella-tibia angle)

In distinguishing between complete ACL tear and intact ACL, the ROC curves indicated that ACL angle had the highest AUC of 0.906 (95% CI 0.833–0.978) followed by PTA with AUC of 0.849 (95%CI: 0.763–0.936), which was close to the AUC of D (0.840; 95% CI: 0.733–0.946). The AUC of FTA was 0.809 (95% CI 0.710–0.908) (Fig. 5A). However, there were no differences among these measurements (*p* > 0.05). In this cohort, the calculated cutoff of ACL angle was 47 degrees with a sensitivity of 90% and specificity of 79%, while the cutoff of D was 4 mm with a sensitivity of 82% and specificity of 81%. For the measurements introduced in the present study, the cutoff of FTA was 80 degrees with a sensitivity of 82% and specificity of 68%, while the cutoff of PTA was 91 degrees with a sensitivity of 82% and specificity of 74%.

To distinguish partial ACL tear from intact ACL, the ROC curves showed that FTA and PTA had the highest AUCs of 0.847 (95% CI: 0.737–0.957) and 0.813 (95% CI: 0.680–0.947), respectively. In contrast, the AUCs of ACL angle and D were only 0.519 (95% CI: 0.292–0.745) and 0.387 (95% CI: 0.227–0.546) respectively, much lower than that of FTA and PTA (*p* < 0.01) (Fig. 5B). However, there was no difference between the AUCs of FTA and PTA (*p* > 0.05). In this cohort, the calculated cutoff of FTA was 84 degrees with a sensitivity of 90% and specificity of 81%, while the cutoff of PTA was 92 degrees with a sensitivity of 80% and specificity of 77%.

Through the study, FTA and PTA were measured in MRI of 160 patients. In the measurements, the concordance rates between two observers were 93.75% and 91.88% for FTA and PTA respectively (differences between the angles were less than 5 degrees). The standard errors were 0.83 and 0.84, respectively. The ICC value was 0.945 (95% CI: 0.918–0.963) for FTA and 0.940 (95% CI: 0.911–0.960) for PTA. Correlation analysis was performed between FTA and PTA through Pearson correlation coefficient (PCC) test. The PCC value was 0.646 (*p* < 0.001) which indicated that FTA and PTA had significant correlation.

## Discussion

The present study introduced two MRI measurements, FTA and PTA, which reflected the rotation of the knee joint and performed well in ACL tear diagnosis. Rotation is an important alteration in ACL deficiency knees [11]. Studies have reported that ACL deficiency could cause significant anterior tibial subluxation and internal tibial rotation at low flexion angles [22]. In the present study, similar results were also found in MRI imaging. In the ACL group of matched comparisons, distance of anterior tibial subluxation was about 2 mm more than that of the control group. For internal tibial rotation, FTA in ACL group was about 5 degrees more than that of isolated meniscus injury group, while PTA was 7 degrees more.

Rotatory laxity of knee joint is also a sign of ACL injury [11, 22]. The unique geometry of the lateral femoral condyle and tibial plateau, combined with the compressive force of the iliotibial band, was shown to contribute to the sudden reduction movement in patients with ACL tears. The most frequently used assessment is pivot shift test, with a reported sensitivity of 49% and a specificity of 98% [23]. Recent study also reported a stepwise increase in quantitative pivot shift examination in terms of rotation with partial ACL tears and complete ACL tears [24]. However, there was few relative quantitative indicators in MRI imaging to assess the rotation of knee joint.

The present study introduced two quantitative indicators, FTA and PTA, to measure the rotation of femur and patella based on the tibia in MRI imaging. In femur and patella, the reference line of the posterior femoral condyles and long axis line of patella were used as previously described [25]. In tibia, because of the heterogeneity of the proximal cortical bone, the posterior parts of tibia or fibula were not suitable as the reference line. In contrast, patellar tendon insertion in tibia appeared as a symmetrical arc or straight line in the coronal plane, which was consistent with the tibia rotation and easy to define at the level of midsagittal plane. As a result, the patellar tendon insertion in tibia was used as the anatomical landmark, and the line perpendicular to patellar tendon insertion was defined as the reference line. In isolated meniscus injury group, the average FTA was 80.91 degrees with SD of 6.11 degrees, and the average PTA was 85.34 degrees with SD of 6.31 degrees. In ACL tear with meniscus injury group, the average FTA was 85.70 degrees with SD of 6.60 degrees, and the average PTA was 92.70 degrees with SD of 5.32 degrees. The concordance between different observers was ideal. Our results showed consistency and reliability of the two measurements.

As for diagnostic performance, FTA and PTA showed diagnostic value in diagnosing ACL tears, especially in distinguishing partial ACL tear. In distinguishing complete ACL tear from intact ACL, although the ACL angle had the highest AUC in the ROC test, it had its limitations. However, in the present study, there were 22.50% (9/40) ACLs in matched comparisons and 30.00% (36/120) ACLs in diagnostic test that cannot be clearly showed in sagittal plane of MRI, which limited its application in MRI. However, FTA or PTA did not have such problem and also provided steady quantitative parameters to assess the rotatory instability that complemented the anterior tibial subluxation parameter.

In terms of distinguishing partial ACL tear from intact ACL, FTA and PTA showed unique advantages compared with ACL angle or D. It remains difficult to confirm partial ACL tears in MRI. Studies have reported that the positive diagnostic value of MRI for the diagnosis of partial ACL tears was less than 50% [26]. Some studies even showed that there was no close correlation between MRI findings and arthroscopically confirmed partial ACL tear [27]. In the present cohort, FTA had a sensitivity of 90% and specificity of 81% while PTA had a sensitivity of 80% and specificity of 77%, which performed well in distinguishing partial ACL tear from intact ACL.

In the current study, the optimal cutoff value was determined using the maximal Youden index. In distinguishing complete ACL tear from intact ACL, the cutoffs of FTA and PTA were 80 and 91 degrees respectively; while in distinguishing partial ACL tear from intact ACL, the cutoffs of FTA and PTA were 84 and 92 degrees respectively. It is interesting that the cutoff degrees in distinguishing partial ACL tear were greater than that in distinguishing complete ACL tear. It is possible that the acute or chronic injury of ACL might play an important role. In complete ACL tears, most of the cases are acute injury with pain and swelling of the knee, which would restrict the knee rotation because of guarding. In contrast, most cases of partial ACL tears are chronic injury without complains and diagnosed by arthroscopy. As for the anterior drawer test, the sensitivity and specificity were also shown to be dependent on the type of the injury [28]. In acute injuries, the sensitivity was only 49% and specificity was 58%, whereas in chronic injuries the results were 92% and 91%, respectively. The changes of soft tissue around the knee after ACL tears may also affect the rotatory laxity. It has been reported that a significant elongated medial collateral ligament and shortened lateral collateral ligament were found after ACL injury [29]. It was more obvious in the chronic ACL tears which would increase the rotation of knee.

The present study introduced two indicators which can be used not only in measuring the rotation of knee joint, but also in diagnosing ACL tear, especially partial ACL tears. The current study has some limitations. Firstly, because of the relative low prevalence of partial ACL tears (10–27% of all ACL injury), only 11 partial ACL tear cases were found in the cohort of 120 consecutive adult patients, which may affect the accuracy of the cutoff value [30]. Another limitation was that the retrospective study design, a well-designed prospective study with adequate sample size is needed to confirm our findings, and validate the value of FTA and PTA in ACL injury, as well as in functional evaluation of ACL reconstruction. Thirdly, although the MRI scans were performed in the supine position with relaxed quadriceps, the measurements might also be affected by the sports activity or muscles features of patients [31, 32]. Lastly, it had indicated that FTA and PTA had significant correlation. However, there were no other angles which can help to guarantee the accuracy of FTA or PTA and it might lead to some bias in the measurements.

## Conclusion

The present study introduced two quantitative parameters, FTA and PTA, to assess the rotation of knee joint. FTA and PTA increased significantly in ACL tears and performed well in diagnostic test. They could be valuable in diagnosing ACL tears, especially in distinguishing partial ACL tear with intact ACL, which may decrease misdiagnosis rate and help preoperative treatment decision-making.

## Data Availability

The datasets used and/or analyzed during the current study are not publicly available due to privacy or ethical restrictions but are available from the corresponding author on reasonable request.

## Abbreviations

FTA: Femur-tibia angle
PTA: Patella-tibia angle
AUC: Area under the curve
ROC: Receiver operating characteristic curve
ACL: Anterior cruciate ligament
MRI: Magnetic resonance imaging
BMI: Body mass index
PACS: Patient archiving and communication system
LPFA: Lateral patellofemoral angle
SD: Standard deviation
CI: Confidence interval
ICC: Interclass correlation
PCC: Pearson correlation coefficient
